# Mobile “doctors” and their medical diagnosis in rural Southern Nigeria. Truth or deception? A public health case report

**DOI:** 10.11604/pamj.2014.17.148.3777

**Published:** 2014-02-28

**Authors:** Chukwuemeka Anthony Umeh, Stella Chioma Onyi, Hycienth Peterson Ahaneku

**Affiliations:** 1 Boston University School of Public Health, Massachusetts, USA; 2 New York Institute of Technology, College of Osteopathic Medicine, New York, USA; 3 Department of Epidemiology, University of Texas School of Public Health, Houston, Texas, USA

**Keywords:** Mobile doctors, traditional healers, herbal medical practitioners, Nigeria

## Abstract

Mobile “doctors” are traditional herbal medical practitioners who move from one rural community in Nigeria to another diagnosing disease using a digital thermometer and stethoscope before selling their herbal drugs to the patients. Are their diagnosis correct or just a deception? This reports looks at three cases of mobile doctors ‘ diagnosis of patients in rural southern Nigeria.

## Introduction

Mobile “doctors” as they are commonly referred to in rural communities in South-South Nigeria are traditional medical practitioners who move from one community to another diagnosing illnesses and treating patients. They use herbal medicine to cure diseases but first they make diagnosis for the patients before prescribing the drugs such patients will take or the procedures that need to be performed for the patient. Such “testing of the body” is usually free and patients have the option of choosing to buy the drugs prescribed or not. The use of herbal medicine has served the health needs of people in Africa for several generations before the introduction of orthodox medicine [1]. The use of herbal medicine is part of the rich African traditional medicine which involves other things such as spiritualism, divination, bone setting etc[1]. In some Asian and African countries, it is estimated that 80% of the population depend on traditional medicine for primary health care and herbal medicines are the most lucrative type of traditional medicine globally [2]. Traditional medicine has been used to treat different infectious and non-infectious diseases and the new antimalarial drug artemisinin was isolated from Artemisia annua L., a plant used in China for almost 2000 years [2, 3, 4]. With the advent of conventional medicine and formal education, people are interested in having a healthcare provider diagnose their illness by either physical examination or laboratory tests before treatment. This might have led to the mobile doctors also progressing to provide free consultation and diagnosis for their patients. However, the consultations and diagnosis provided by these traditional mobile doctors are unique. They do not ask their patients what is wrong with them (no history taking). The patients just come and they examine them and tell the patients what the problem is. Interviewing some of the people revealed that these people diagnose the problem using a digital thermometer and a stethoscope. They put the thermometer in the anterior part of the elbow joint and tell the patient to flex their elbow joint. Some of them also use the stethoscope to touch different parts of the body and after then tell the patient what is wrong with him/her. The cases below are patients that one of the authors encountered while living and working in hospitals in rural communities in Bayelsa state Nigeria.

## Patient and observation

**Case 1:** There was a middle aged mother of three who married a new husband and wanted to raise a family with the new husband but had not succeeded after five years. A mobile “doctor” tested her using a digital thermometer and a stethoscope and told her that the blood did not go out completely after her last delivery 10 years earlier and that is why she was having problems. He told her that the retained blood is the reason why she has waist pain, back pain, chest pain and headache and that the blood turns as if it is a child and when it turns she feels chest pain and dizzy. Furthermore, he said that the retained blood has made her womb to shift and that if she does not treat the condition the blood can turn to fibroids and cause her to have irregular or heavy mensuration. He told her that she needs to pay for some drugs to take and after that she will go for evacuation of the womb in a hospital before she can become pregnant. The woman bought the drugs and took them and after that went to see a gynecologist and requested for “washing of the womb”. Ultrasound result showing that there was no retained blood in the uterus was not enough to convince the woman especially after a second mobile doctor had examined the woman using a stethoscope and told her that her womb is hot and that it is the hotness of the womb that is making her not to conceive.

**Case 2:** There was another case of a 47 years old man who smokes. He was attending clinic and was constantly counseled on the need to stop smoking and the resources available to him to help him stop. He was working on what the best strategy to use to quit smoking until he met one mobile doctor who just came into town. The mobile doctor examined him with a digital thermometer and told him that the heart and lungs were looking black and asked him if he smokes, to which he answered in the affirmative. The mobile doctor said that he was going to sell him drugs that will wash the heart and lungs and remove the black covering that resulted from smoking. Just because the mobile “doctor” was a total stranger in the town who did not know that the man smokes, the man believed all that he told him and bought the drugs at an expensive price and dropped the idea of quitting smoking. Several efforts to convince the man that the drugs are not working proved abortive and the man died years later from a diagnosis of lung cancer.

**Case 3:** Another man gave an account of his family ‘s encounter with a mobile “doctor” who came to their house to test and diagnose them. He said that being a bit literate; he was not interested in the diagnosis but that the wife opted to have the mobile “doctor” examine her. After putting the digital thermometer on her elbow as usual, he told her that she has hypertension and that she has hot feelings in her abdomen. The “doctor” said that the hot feeling in her abdomen is because there are some fluids in her abdomen and that her fallopian tubes are blocked. He said that she should bring 8,000 naira ($50) so that he could give her drugs to flush open the fallopian tubes so that the fluid in the abdomen will go and that the hot feeling in the abdomen will stop. The man said that what got him surprised and confused was that the wife was a known hypertensive. Then when he asked the wife if she has a feeling of abdominal hotness, the wife answered in the affirmative. So he was wondering how someone could make such accurate diagnosis with just a digital thermometer. Although, they did not buy the drugs, it made him to believe that perhaps the mobile “doctors” could be right after all.

## Discussion

The use of digital thermometers and stethoscope by mobile doctors to make diagnosis in rural communities in south-south Nigeria makes healthcare practice difficult for conventional health care providers practicing in these rural communities. This is because patients walk into the clinic and see the healthcare providers using the same thermometer and stethoscope that the mobile doctors use and when the patients are asked what their problems are, they tell the healthcare providers to test them and find out what is wrong with them just as the mobile doctors do. Even when health care providers explain to them that they need to say how they are feeling first so that the physical and laboratory investigations will be focused, some of the patients go home with the impression that the health care providers do not know their jobs. This is because most patients in rural Nigeria already believe that doctors should know all diseases [5], and should at least be more knowledgeable than the mobile doctors. The big question is how the mobile doctors can be making all their diagnosis by just using a thermometer and stethoscope. One option might be that the mobile doctors work on patients ‘ psychology. They may just mention common symptoms which they know people normally have and then begin to explain why the person is having the symptom. Due to the fact that most times they correctly mention patients ‘ symptoms without the patients telling them, it is easy for the patients to believe everything that they tell them thereafter.

However, another school of thought believes that the mobile doctors use spiritual power and divination to know the problems of the patients. Over the years traditional medicine practitioners have been known to use a combination of different methods - herbs, divination, spiritualism etc. With divination, it is possible to know the cause of an illness which can then be subsequently treated[1, 6, 7]. With the advent of Christianity in southern Nigeria, African traditional divination and spiritualism is considered as demonic. This makes modern African traditional healers to practice in such a way that the spiritual aspects of their practice will not be called into question. Thus, the use of digital thermometers and stethoscope might just be a cover up from making people feel that they are using spiritual divination in making the diagnosis.

It is important to note that the majority of these mobile doctors practice in rural communities where people are uneducated or poorly educated and are more gullible to their operations. There is need to protect this vulnerable population from the practice of these mobile doctors. It is better for the mobile doctors to clearly come out and sell their herbs instead of deceiving the population. The Nigerian National Agency for Food Administration and Control (NAFDAC) have granted marketing authorization to over 1,000 well researched traditional medicinal products for various diseases such as malaria, diabetes, sickle cell diseases and hypertension[8]. However, there is need to regulate the way these products are marketed to ensure that the population is protected.

## Conclusion

In as much as some of the medical diagnosis by these traditional mobile doctors might be correct, the method through which they make the diagnosis is wrong and a deception and should be stopped. There is need to not only regulate the production of traditional medicine in Nigeria, but there is also urgent need to regulate the way these drugs are being marketed to patients especially the poorly educated vulnerable population in rural communities.
